# Ultra-Fast Image Reconstruction of Tomosynthesis Mammography Using GPU

**Published:** 2015-06-01

**Authors:** D. Arefan, A. Talebpour, N. Ahmadinejhad, A. Kamali Asl

**Affiliations:** 1 Medical Radiation Department, Shahid Beheshti University, Tehran, Iran; 2 Electrical and computer engineering, Shahid Beheshti University, Tehran, Iran; 3 Tehran University of medical science, Imam Khomeini hospital ADIR, Tehran, Iran

**Keywords:** Breast, Graphics Processing Unit, Image Reconstruction, Mammography, Tomosynthesis

## Abstract

Digital Breast Tomosynthesis (DBT) is a technology that creates three dimensional (3D) images of breast tissue. Tomosynthesis mammography detects lesions that are not detectable with other imaging systems. If image reconstruction time is in the order of seconds, we can use Tomosynthesis systems to perform Tomosynthesis-guided Interventional procedures. This research has been designed to study ultra-fast image reconstruction technique for Tomosynthesis Mammography systems using Graphics Processing Unit (GPU). At first, projections of Tomosynthesis mammography have been simulated. In order to produce Tomosynthesis projections, it has been designed a 3D breast phantom from empirical data. It is based on MRI data in its natural form. Then, projections have been created from 3D breast phantom. The image reconstruction algorithm based on FBP was programmed with C++ language in two methods using central processing unit (CPU) card and the Graphics Processing Unit (GPU). It calculated the time of image reconstruction in two kinds of programming (using CPU and GPU).

## Introduction

Digital Breast Tomosynthesis (DBT) is a technology that creates three dimensional (3D) images of breast tissue. It is utilized in numerous countries outside the United States of America since 2009. Food and Drug Administration of U.S.A. (FDA) has approved the Hologic Selenia Breast Tomosynthesis System in February 2011[1]. Tomosynthesis mammography detects lesions that aren’t detectable with other imaging systems. If image reconstruction time is in the order of seconds, we can use Tomosynthesis systems to perform Tomosynthesis-guided Interventional procedures. A biopsy procedure entails 3 steps; scout, pre-fire and post-fire scans. So, rapid scans and high speed image reconstruction are very important in these systems. To improve reconstruction speed, we can take advantage of the computational power of modern graphics processing units (GPUs)[2]. This research has used GPU for the accelerating of image reconstruction’s time. There are several methods for Tomosynthesis image reconstruction but Filtered Back-Projection (FBP) has been commonly used as an efficient and robust reconstruction technique in tomographic X-ray imaging during the last decades[3]. In this research, generalized filter back projection (FBP), algorithm proposed by Klaus Erhard, Michael Grass and Sebastian Hitziger for Tomosynthesis image reconstruction were utilized[3]. Materials and methods of this research are described in section 2. In this section, it has been described the method of image reconstruction and creating 3D digital breast phantom and also it has been shown how x-ray projections are produced from phantom. Results and comparison between CPU and GPU programming have been presented in Section 3 and conclusions have been presented in Section 4. 

## Material and Methods

### Filter Back Projection

Filter Back Projection (FBP) is one of the best algorithms for image reconstruction in tomographic X-ray imaging but in Tomosynthesis mammography, we do not have a complete source trajectory and projections. Because of this, in this research a generalized 2FBP algorithm proposed by Klaus Erhard, Michael Grass and Sebastian Hitziger for Tomosynthesis image reconstruction is used[4].

### GPU

**Figure 1:**
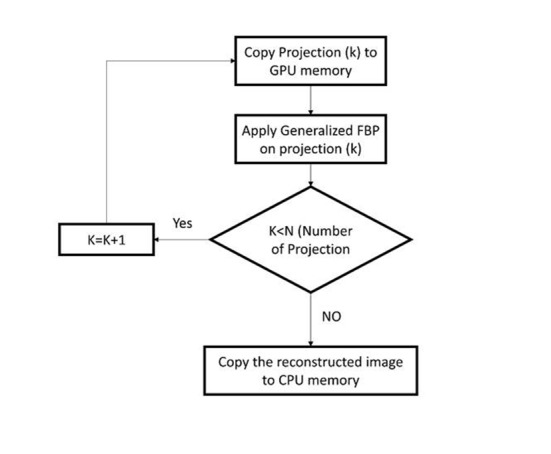
GPU programming block diagram

Graphic Processing Unit (GPU) is a processor that was specialized for processing graphics and has recently evolved towards a more flexible architecture. The GPU handles parallel execution and thread management. The host OS does not have to do anything and everything is managed by the GPU driver. In this research, Nvidia’s CUDA framework for GPU programming has been used. The CPU card consisted of 4 processing cores with 2.4 GHz clock speed and 4096 MB RAM on a 64-bit Linux OS and our GPU unit consisted of 64 stream processors with 650 MHz core clock and 512 MB of GDDR3. Figure 1 shows the general GPU programming block diagram.

### Breast Phantom

The size and density of breast tissue are very variable from person to person. Much work has been done to generate a 3D realistic breast phantom. Zhou et al. introduced some work to generate a 3D breast phantom[4]; in one study, they generated a 3D breast phantom composed of large scale structures  odelin by two ellipsoidal regions representing adipose tissue and fibrous-glandular tissue, and medium scale structures  odelin by spherical shells, blobs, and ductal trees. A statistical model of ductal tree generation was proposed. A crescent-shaped fibro-glandular region was formed along with a sharp and regular margin in the resulting synthetic mammogram[5]. In another study, a methodology for 3D breast odeling was presented. The simulated breast includes some major tissue features. Their model consists of overall breast shape, adipose tissue, duct system, Cooper’s ligaments, abnormalities, pectoral muscle, and mammographies background. Their model included random generation of variable structures[6]. Li et al. presented a methodology for generating a 3D computerized breast phantom from empirical data. They have used breast CT data acquisition and image processing techniques like segmentation, region growing, noise reduction, scatter correction and so on to generate a 3D breast phantom[7]. In this research, a 3D computerized breast phantom from empirical data has been generated based on MRI data in its natural form. Breast MRI image was used in this research obtained from researches at Ghaem MRI Center in Mashhad, Iran by Siemens-Avanto MRI system reconstructed in 3D space. Table 1 shows the imaging data in detail. Breast MRI is performed with a dedicated breast imaging coil and the patient is in a horizontal position on a scanning table with the suspended breast through an aperture in the table of MRI system without compression like breast CT imaging system[4]. Hsu et al. presented a technique that can be used to generate a suite of realistic computerized breast phantoms from a limited number of human subjects[8]. The purpose of this research is to compare GPU and CPU programming. Detailed anatomy in breast phantom isn’t very important. For this reason, correction of scatter, noise and any segmentation technic are unnecessary. Just on the background of MRI, 3D breast image is the only problem because it causes to produce false objects in projections. For detecting and removing background and extracting breast in MRI 3D image, Otsu method in 3D space has been used.

**Table 1:** The measured uncertainty values in personal dosimetry by TLD.

**Modality**	**Manufacturer**	**Model**	**Patient** **Age**	**Patient** ** Weight**	**Acquisition** **Type**	**Slice** **Thickness**	**Software** **Version**	**Image** **Size**	**Image** **Depth**
MR	Siemens	Avanto	39	85 Kg	3D	1.1 mm	Syngo MR B17	384*384	12

#### Otsu method

Otsu’s method is named after Nobuyuki Otsu and is used to automatically perform clustering-based image thresholding. According to Otsu method, the threshold that minimizes intra-class variance is the best threshold for separating background and foreground in images:

$$\sigma_{w}^{2}\left(t\right)=w_{1}\left(t\right)\sigma_{1}^{2}\left(t\right)+w_{2}\left(t\right)\sigma_{2}^{2}\left(t\right)$$

*w*_1_(*t*) and *w*_2_(*t*) are  the probabilities of the two classes separated by a threshold *t* and σ_1_^2^(t) and σ_2_^2^(t) variances of these classes. Otsu shows that minimizing the intra-class variance is the same as maximizing inter-class variance[9]:

$$\sigma_{b}^{2}\left(t\right)={w_{2}\left(t\right)[{µ}_{1}\left(t\right)-{µ}_{2}\left(t\right)]}^{2}$$

As:

$$w_{1}\left(t\right)=\sum_{0}^{t}P_{i}\;\;And\;w_{2}\left(t\right)=\sum_{r+1}^{n}P_{i}$$

μ_1_(*t*) and μ_2_(*t*) are computed:

$${µ}_{1}\left(t\right)=[\sum_{0}^{t}P_{i}X_{i}]/w_{1}\;\;And\;{µ}_{2}\left(t\right)=[\sum_{r+1}^{n}P_{i}X_{i}]/w_{2}$$

**Figure 2:**
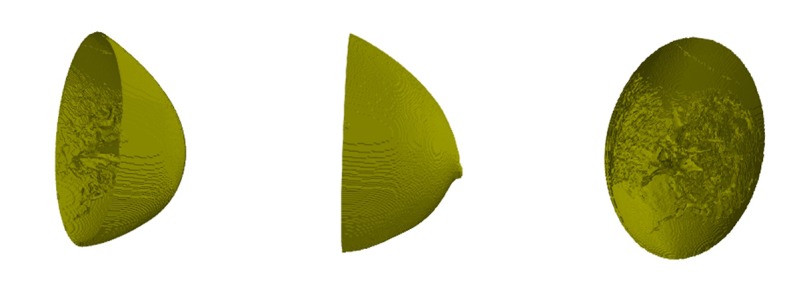
3D digital phantom in different views

In this research, the best threshold for separating foreground and background by Otsu method and plotting histogram of MRI breast image in 3D space was calculated in order to remove background and extract breast image. At last, the computerized 3D breast phantom was imported to MATLAB software for creating x-ray projections at different angles. In figure 2 the 3D digital phantom has been shown.

### Projections

**Figure 3:**
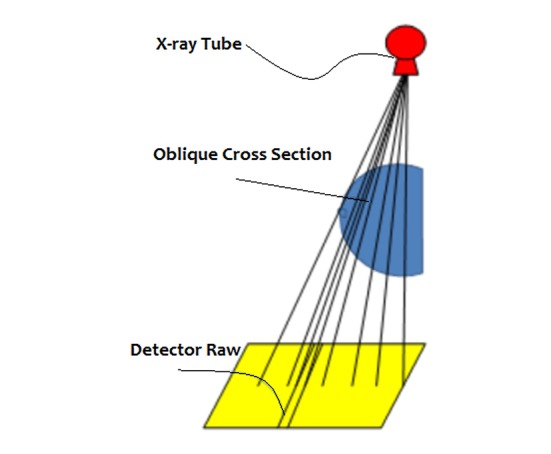
Oblique cross sections in breast phantom

In order to produce x-ray projection from breast phantom, we used MATLAB (7.14.0.739) software. For simulating oblique x-rays, 2D oblique cross sections in breast phantom were calculated such as mammography x-ray model and projections for each row of detectors have been produced by sum of the attenuation number of each oblique surface. The purpose of this research is to compare speed of CPU and GPU programming in image reconstruction of Tomosynthesis mammography. To serve this purpose, any kind of noise from projections was removed. Figure 3 shows the oblique cross-sections in breast phantom.

## Results

We created 15 projections from 3D breast phantom sized 256*256 pixels from 15-degree scan angle (-7.5 to +7.5 degrees) similar to Hologic Selenia Dimensions Breast Tomosynthesis system. We used generalized filter back projection (FBP) algorithm proposed by Klaus Erhard, Michael Grass and Sebastian Hitziger for Tomosynthesis image reconstruction. The image reconstruction algorithm based on generalized FBP was programed with C++ language in two methods using Central Processing Unit (CPU) card and Graphics Processing Unit (GPU). The CPU card consisted of 4 processing cores with 2.4 GHz clock speed and 4096 MB RAM on a 64-bit Linux OS and our GPU unite consisted of 64 stream processors with 650 MHz core clock and 512 MB of GDDR3. We calculated the time of image reconstruction in two kinds of programming (using CPU and GPU). The image reconstruction time was 9s and 1s using CPU and GPU programming, respectively. Table 2 shows the results in detail.

**Table 2:** Comparison of the CPU hardware to GPU hardware in image reconstruction time

**Hardware**	**Reconstruction** **Time(sec)**	**Reconstruction** **Algorithm**	**Performance**	**OS**	**RAM(MB)**
CPU	9.03	FBP	2.4 GHz- 4 cores	64-bit Linux	4096
GPU	1.01	FBP	650 MHz – 64 processors	64-bit Linux	512 MB of GDDR3

## Conclusion

In this research, the speed of image reconstruction in two kinds of CPU and GPU programming has been compared to indicate that GPU programming is about 9 times faster than CPU programming. The speed of image reconstruction is very important for using Tomosynthesis systems to perform Tomosynthesis-guided Interventional procedures. Using GPU programming in an image reconstruction algorithm can be useful for this purpose. 

## References

[1] Smith A The Use of Breast Tomosynthesis in a Clinical Setting [Internet].

[2] Schiwietz T, Chang T, Speier P, Westermann R, Flynn MJ, Hsieh JI (2006). MR image reconstruction using the GPU. Physics of Medical Imaging Proceeding.

[3] Erhard K, Grass M, Hitziger S, Iske A, Nielsen T, Pelc NJ, Nishikawa RM, Whiting BR (2012). Generalized filtered back-projection for digital breast tomosynthesis reconstruction. Physics of Medical Imaging Proceeding.

[4] Zhou L, Oldan J, Fisher P, Gindi G, Flynn MJ, Hsieh JI (2006). Low-contrast lesion detection in tomosynthetic breast imaging using a realistic breast phantom. Physics of Medical Imaging Proceeding.

[5] Bakic PR, Albert M, Brzakovic D, Maidment AD (2002). Mammogram synthesis using a 3D simulation. I. Breast tissue model and image acquisition simulation. *Med Phys*.

[6] Bliznakova K, Bliznakov Z, Bravou V, Kolitsi Z, Pallikarakis N (2003). A three-dimensional breast software phantom for mammography simulation. *Phys Med Biol*.

[7] Li CM, Segars WP, Tourassi GD, Boone JM, Dobbins JT 3rd (2009). Methodology for generating a 3D computerized breast phantom from empirical data. *Med Phys*.

[8] Hsu CM, Palmeri ML, Segars WP, Veress AI, Dobbins JT 3rd (2013). Generation of a suite of 3D computer-generated breast phantoms from a limited set of human subject data. *Med Phys*.

[9] Otsu N (1975). A threshold selection method from gray-level histograms. *Automatica*.

